# Laryngospasm Treated With Intramuscular Rocuronium in a Pediatric Patient Without Intravenous Access: A Case Report

**DOI:** 10.7759/cureus.56620

**Published:** 2024-03-21

**Authors:** Hiroki Tabata, Kenichi Takahoko, Sarah K Luthe, Hiroshi Makino, Hajime Iwasaki

**Affiliations:** 1 Department of Anesthesiology and Critical Care Medicine, Asahikawa Medical University, Asahikawa, JPN; 2 Department of Anesthesia, Indiana University School of Medicine, Indianapolis, USA

**Keywords:** succinylcholine, rocuronium, pediatric anesthesia, laryngospasm, intramuscular rocuronium

## Abstract

We present a case in which intramuscular rocuronium was used successfully to treat laryngospasm in a pediatric patient. An 11-month-old infant weighing 9.7 kg was scheduled for an elective laparoscopic inguinal hernia repair surgery. Anesthesia was induced with oxygen, nitrous oxide, and sevoflurane. After loss of consciousness, mask ventilation became impossible, and laryngospasm was suspected. Intravenous access was attempted without success; as there was no immediate access to succinylcholine, rocuronium 10 mg (1.0 mg/kg) was injected intramuscularly into the vastus lateralis muscle. We were able to mask-ventilate the patient within one minute of intramuscular rocuronium, followed by successful endotracheal intubation with a video laryngoscope. The duration of hypoxia (saturation of peripheral oxygen (SpO2) < 90%) was approximately two minutes, and the patient's lowest oxygen saturation during induction was 76%. At the end of the surgery, the patient was uneventfully extubated. We conclude that intramuscular rocuronium may provide an alternative treatment for laryngospasm in pediatric patients with no intravenous access and no availability of succinylcholine.

## Introduction

Laryngospasm is a common complication during inhalational induction in pediatric anesthesia. Intramuscular (IM) succinylcholine is the gold standard for treating laryngospasm when the patient has no intravenous (IV) access [1]. However, accessibility and availability of succinylcholine can differ among institutions. Therefore, utilizing rocuronium for IM injection in the pediatric patient population has been investigated as rocuronium is a widely used non-depolarizing neuromuscular blocking agent [2]. However, there is no report of IM rocuronium used successfully to resolve laryngospasm during pediatric anesthesia. We describe a case of laryngospasm in a pediatric patient without IV access treated with IM rocuronium.

## Case presentation

An otherwise healthy 11-month-old male infant weighing 9.7 kg was scheduled for elective laparoscopic inguinal hernia repair surgery. On preoperative evaluation, the patient had no significant past medical history and did not present with any upper respiratory tract symptoms on the day of surgery.

The patient received no premedication. Standard monitors including three-lead electrocardiography, noninvasive blood pressure, and pulse oximetry, were placed prior to induction. Anesthesia was induced with oxygen, nitrous oxide 50-60%, and sevoflurane 1-8%. After loss of consciousness, mask ventilation suddenly became impossible. End-tidal CO2 was undetectable despite the facemask ventilation with 20-30 mmHg peak pressure, and laryngospasm was suspected. Peripheral IV access was attempted without success while providing continuous positive airway pressure with 100% oxygen using a face mask.

Given that the patient was quickly desaturating and due to no immediate access to succinylcholine, rocuronium 10 mg (approximately 1.0 mg/kg) was injected intramuscularly into the vastus lateralis muscle. We were able to mask-ventilate the patient within one minute of administration of IM rocuronium. The endotracheal intubation was successfully performed using a McGRATH™ MAC video laryngoscope (Medtronic, Minneapolis, Minnesota, United States). Visualization of abducted vocal cords was noted on a video laryngoscope image. The duration of hypoxia (SpO2 < 90%) was approximately two minutes. The patient's lowest oxygen saturation during the induction was 76% without bradycardia or other adverse events.

After the endotracheal intubation, acceleromyography (IntelliVue NMT Module, Koninklijke Philips N.V., Amsterdam, Netherlands) was used to monitor neuromuscular block at the adductor pollicis muscle using 30 mA ulnar nerve stimulation without calibration. Anesthesia was maintained with air, oxygen, sevoflurane, and a remifentanil infusion. No additional rocuronium was administered as the train-of-four (TOF) count was zero throughout the surgery. The surgery was completed uneventfully.

**Figure 1:**
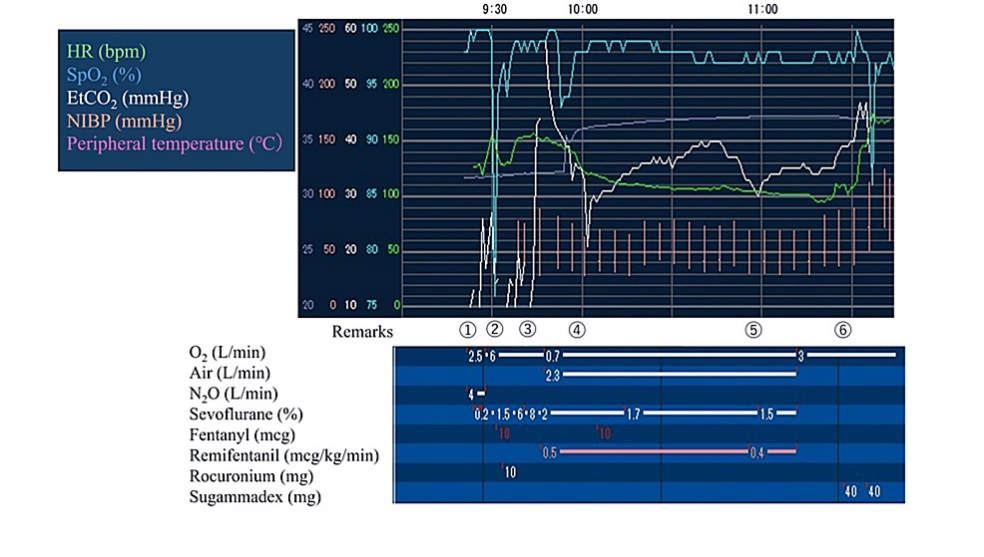
Anesthetic records Remarks: ① Induction ② Intramuscular rocuronium ③ Endotracheal intubation ④ Start of surgery ⑤ End of surgery ⑥ Extubation HR: heart rate; SpO2: saturation of percutaneous oxygen; EtCO2: end-tidal CO2; NIBP: non-invasive blood pressure

At the end of surgery (110 minutes after IM administration of rocuronium), neuromuscular monitoring showed one response to post-tetanic count (PTC) stimulation. After reversing the neuromuscular block with 4 mg/kg sugammadex and confirming the recovery of the TOF ratio to 100%, the patient was uneventfully extubated. Given the potential risk of prolonged duration of action of rocuronium and recurarization due to the IM route of administration, an additional dose of 4 mg/kg sugammadex (a total of 8 mg/kg) was administered after extubation. Anesthetic records are shown in Figure 1. No adverse events were identified throughout the hospital course.

## Discussion

This report describes the successful use of IM rocuronium 1.0 mg/kg in treating laryngospasm in a pediatric patient without IV access. The gold standard to treat laryngospasm during the absence of IV access is IM succinylcholine [1]. It is reported that 4 mg/kg or even smaller doses of IM succinylcholine can quickly relieve laryngospasm [1,3]. However, accessibility and availability of succinylcholine can differ among institutions. In many facilities in Japan, succinylcholine is mandated to be secured in a locked safe, and some require reconstitution with 0.9% sodium chloride or 5% dextrose for administration. It is also used less in the pediatric population to avoid the incidence of bradycardia and triggering malignant hyperthermia. In contrast, rocuronium is drawn up from a vial into a syringe and prepared for most cases planned for endotracheal intubation.

 In regard to the onset of action, our experience shows that IM rocuronium 1.0 mg/kg took only a minute to relieve laryngospasm. According to previous studies, five to seven minutes were required to obtain complete twitch depression with IM rocuronium 1.0 mg/kg in infants [4,5]. Therefore, IM rocuronium is considered unsuitable for endotracheal intubation. However, in managing laryngospasm, a complete neuromuscular block is likely unnecessary to break the laryngospasm and improve oxygenation and ventilation. Moreover, the onset of neuromuscular block is known to be more rapid at the larynx compared to peripheral muscles [6]. This is consistent with the immediate relief of laryngospasm with IM rocuronium witnessed in our case.

The site of IM rocuronium must be considered as a factor influencing the onset. It was previously reported that IM rocuronium administered to the deltoid muscle showed a rapid onset compared to IM rocuronium administered to the leg muscle [2]. Therefore, if rocuronium had been administered to the deltoid muscle, laryngospasm might have been relieved more quickly.

One concern with IM rocuronium is the possibility of prolonged duration of neuromuscular block compared to IV administration. The duration of action of rocuronium might be increased if absorption from the IM site was delayed [5]. According to a study by Kaplan et al., children who received IM rocuronium had a peak plasma concentration at 13 minutes and 80% bioavailability from the IM site [4]. A simulation in the study showed that less than 4% of the administered dose remained to be absorbed from the intramuscular site 30 minutes after injection; therefore, the risk of recurarization is limited if more than 30 minutes elapse from the time of injection and adequate reversal and monitoring. Moreover, none of the patients who received IM rocuronium and reversed with neostigmine experienced residual neuromuscular block [4]. Therefore, based on previous literature, an additional 4 mg/kg sugammadex to avoid recurarization in our case may not be necessary.

Laryngospasm may occur not only during the induction of anesthesia but also during extubating the trachea [1]. As IV access may be available during the extubation phase, the following treatment will be the option: IV succinylcholine 0.1-3 mg/kg with atropine 0.02 mg/kg, propofol 0.25-0.8 mg/kg [7].

## Conclusions

In the event of no IV access and no availability of succinylcholine, IM rocuronium may provide an alternative treatment for laryngospasm in pediatric patients. Careful neuromuscular monitoring and reversal are required as the duration of action of rocuronium may be prolonged when administered IM compared to IV administration.
